# Spread of the *bla*_OXA–23_-Containing Tn*2008* in Carbapenem-Resistant *Acinetobacter baumannii* Isolates Grouped in CC92 from China

**DOI:** 10.3389/fmicb.2017.00163

**Published:** 2017-02-06

**Authors:** Yisheng Chen, Jing Gao, Haomin Zhang, Chunmei Ying

**Affiliations:** ^1^Department of Clinical Laboratory, Obstetrics and Gynecology Hospital of Fudan UniversityShanghai, China; ^2^Department of Clinical Laboratory, Renji Hospital, Shanghai Jiao Tong University School of MedicineShanghai, China

**Keywords:** carbapenem-resistant *Acinetobacter baumannii* (CRAB), *bla*_OXA–23_, transposon, plasmid replicase genes, multilocus sequence type (MLST)

## Abstract

The rapid expansion of carbapenem-resistant *Acinetobacter baumannii* (CRAB) clinical isolates is a big issue. We investigated the antibiotic susceptibility, molecular epidemiology and resistance gene of *A. baumannii* collected at two hospitals in Shanghai, China. Besides, the *A. baumannii* PCR-based replicon typing method (AB-PBRT) was conducted to categorize the plasmids into homogeneous groups on the basis of replicase genes. Most CRAB isolates showed high-level resistance to almost all antibiotics but retain susceptibility to colistin and tigecycline. A total of 101 isolates carried *bla*_OXA-51_-like gene. Sequencing identified the presence of *bla*_OXA-66_ for CRAB isolates. *bla*_OXA–23_ gene were discovered in all CRAB isolates. Each CRAB isolate contained 1–3 of 19 different plasmid replicase (*rep*) gene homology groups (GRs) and the GR6 (*rep*Aci6) was ubiquitous. Genotyping by Multilocus Sequence Typing (MLST) showed seven defined MLST patterns and three novel STs were found. eBURST analysis indicated they were all grouped in CC92 (GCII) with the most frequent ST208 (50%). Two *bla*_OXA–23_-bearing transposons were found: Tn*2006* and Tn*2008*. Tn*2008* were detected in 54 (96.4%) isolates and Tn*2006* in two remaining isolates. The *bla*_OXA–23_ carbapenem gene was vitally associated with *rep*Aci6 plasmid belong to CC92 clonal group. Our survey revealed severe drug resistance in *A. baumannii* isolates. Tn*2008*-containing CC92 *A. baumannii* were endemic, which may facilitate the *bla*_oxa23_ dissemination.

## Introduction

*Acinetobacter baumannii* is a significant opportunistic pathogen responsible for numerous nosocomial infections, including respiratory infections (in particular ventilator-associated pneumonia, VAP), urinary tract infections, bacteremia, soft and skin tissue infections, burn wound infections and secondary meningitis (Roca et al., 2012; Kempf et al., 2013). Carbapenem resistance in *A. baumannii* is an emerging problem worldwide during the last decade (Jiang et al., 2014; Guerrero-Lozano et al., 2015). One data from Taiwan involving five major hospitals showed that resistance to imipenem in intensive care units increased from 22.0% in 2000 to 66.8% in 2010 (Hsueh et al., 2001). Studies performed by Reddy also reported a disturbing tendency of sharp increase in the rates of CRAB isolates, from 1% in 2003 to 58% in 2008 (Reddy et al., 2010). Data from Wallace also found the percentage of CRAB isolates was 31% before the year 2009 followed by 99% after the year 2009 at the University of Maryland Medical Center (UMMC; Wallace et al., 2016).

Carbapenem-resistant in *A. baumannii* is due to combined mechanisms including production of OXA- and metallo-β-lactamases, outer membrane impermeability, increased expression of eﬄux pumps, and penicillin-binding proteins modification (Zarrilli et al., 2013). OXA-type enzymes related to carbapenem resistance include the natural *bla*_OXA-51_-like and three acquired genes: *bla*_OXA–23_-like, *bla*_OXA-24_-like and *bla*_OXA-58_-like (Jiang et al., 2014). These genes were integrated in the bacterial chromosome or carried by plasmids (Poirel et al., 2010). Nowadays, AB-PBRT method provides a new tool to investigate the epidemiology of plasmids in *A. baumannii* (Towner et al., 2011). Numerous publications reported the spread of resistance gene via transposons (Poirel et al., 2010). Four transposons harboring *bla*_OXA–23_ gene have been reported: Tn*2006*, Tn*2007*, Tn*2008*, and Tn*2009* (Zhou et al., 2011; Espinal et al., 2013; Guerrero-Lozano et al., 2015). These types of transposons share a common region “*bla*_OXA–23_-ΔATPase.” Tn*2007* owns IS*Aba4* promoter upstream *bla*_OXA–23_ gene. The remaining three transposons own IS*Aba1*. The two IS*Aba1* copies were inversely orientated in Tn*2006* compared to the same direction in Tn*2009* (Guerrero-Lozano et al., 2015). Tn*2006* and Tn*2008* are reported globally disseminated, while Tn*2009* has only been discovered in China (Zhou et al., 2011). These IS*Aba1*-associated transposons contributed to the dissemination of *bla*_OXA–23_. Moreover, Tn*2006*, Tn*2008*, and Tn*2009* all have been found in conjugative plasmids except Tn*2007*. It is noted that Tn*2007* is immovable and is not actually considered as a transposon (Nigro and Hall, 2016).

Colistin is currently used as last-resort antibiotics against CRAB infection (Durante-Mangoni et al., 2014). It is important to analyze the local epidemiology of clinical CRAB isolates and control the dissemination. The aim of the present study was to investigate the drug-resistance and molecular characteristics of *A. baumannii* isolates in 101 *A. baumannii* clinical isolates from two hospitals in Shanghai. We also characterized the genetic environment surrounding *bla*_OXA–23_ gene. In addition, the distribution and epidemiology of plasmid replicase (*rep*) genes in CRAB isolates were also investigated.

## Materials and Methods

### Bacterial Isolates

A total of 101 non-repetitive *A. baumannii* clinical isolates were collected in this study from two hospitals in Shanghai, China. All subjects were anonymised in this study. Eighty-seven isolates were recovered from neurosurgery in Renji Hospital Shanghai Jiaotong University School of Medicine from July 2011 to June 2013. And the remaining 14 isolates were collected from Obstetrics and Gynecology Hospital of Fudan University from January 2015 to August 2016. All isolates were identified using the VITEK 2 Compact automatic bacteria and drug susceptibility analysis system.

### Antimicrobial Susceptibility Testing

Antibiotic susceptibility testing was conducted in the present study. Fourteen antimicrobial agents were tested including piperacillin/tazobactam, cefoxitin, ceftazidime, cefotaxime, imipenem, meropenem, gentasin, amikacin, minocycline, ciprofloxacin, trimethoprim/sulfamethoxazole, cefepime, polymyxin, and tigecycline. The Minimum inhibitory concentrations (MICs) were determined by agar dilution method except polymyxin, which was performed by broth dilution method. *Pseudomonas aeruginosa* ATCC 27853 and *Escherichia coli* ATCC 25922 were used as reference strains. The susceptibility results were interpreted based on the Clinical and Laboratory standards Institute (CLSI) breakpoints (CLSI, 2011). For tigecycline, we used breakpoints recommended by the British Society for Antimicrobial Chemotherapy guidelines (BSAC, 2011). CRAB isolates were defined with both imipenem and meropenem resistance (MICs > 8 mg/L).

### PCR and Sequencing of Drug Resistance Genes

PCR assay was performed to screen the presence of drug resistance genes in 101 isolates, including *bla*_OXA–23_-like, *bla*_OXA-24_-like, *bla*_OXA-51_-like, *bla*_OXA-58_-like, *bla*_IMP-1_, *bla*_V IM-1_, *bla*_V IM-2_, and *bla*_AmpC_. The entire products were sequenced and analyzed with the BLAST website^1^.

### Multilocus Sequence Typing

Molecular typing of CRAB isolates was determined by MLST. Briefly, it was detected by sequence analysis of gltA, gyrB, gdhB, recA, cpn60, gpi, and rpoD housekeeping genes as previously described (Ying et al., 2015). Primers are available at http://pubmlst.org/abaumannii/. The results were compared with the STs databases online at http://pubmlst.org/databases/. eBURST analysis was conducted to investigate the genetic relationships and clonal complexes (CCs) of these isolates.

### Genetic Environment of the *bla*_OXA–23_ Gene

**FIGURE 1:**
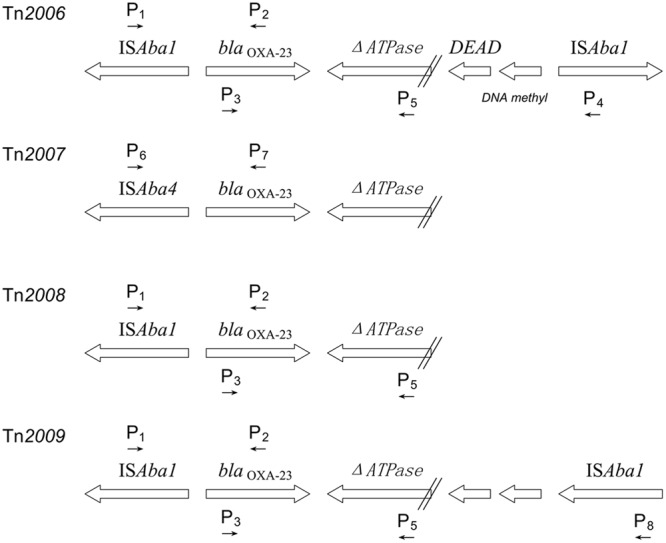
**Structures and primer locations of Tn*2006*, Tn*2007*, Tn*2008*, and Tn*2009***.

PCR was performed to detect the occurrence of *bla*_OXA–23_-carrying transposons in CRAB isolates, including Tn*2006*, Tn*2007*, Tn*2008*, and Tn*2009*. Primers used in this study were shown in **Table 1**. Structures and primer locations were shown in **Figure 1**. In addition, *bla*_OXA–23-_*ΔATPase* was the common region of Tn*2006* and Tn*2008*.

**Table 1:** Primers used for *bla*_OXA–23_ gene detection.

Primer name	Primer sequence (5′ –3′)	Replicase gene
Tn*2006* Int-P3	GTCTATCAGGAACTTGCGCG	IS*Aba1-bla*_OXA–23_-IS*Aba1*
Tn*2006* Int-P4	GCAAGGCTTTAGATGCAGAAGA	
Tn*2007* Int-P6	ATTTGAACCCATCTATTGGC	IS*Aba4*- *bla*_OXA–23_
Tn*2007* Int-P7	ACTCTCATATTTTTTCTTGG	
Tn*2006/8* Int-P3	GTCTATCAGGAACTTGCGCG	*bla*_OXA–23-_*ΔATPase*
Tn*2006/8* Int-P5	GGCTCATTACAGTCAGGTACAAGT	
Tn*2009* Int-P1	ATCCTGATGCTCGCAATCGT	IS*Aba1-bla*_OXA–23_-IS*Aba1*
Tn*2009* Int-P8	CTGTCTGCGAACACATTCAC	

### AB-PBRT

Nineteen PCR amplifications were organized into six multiplexes and used to detect 27 different plasmid *rep* genes as described previously (Bertini et al., 2010). Each PCRs recognized three or four different homology groups (GRs). PCR products were recognized by DNA sequencing. An additional PCR was performed to identify the gene encoding the type IV secretion system protein TraC, which was found on the plasmid pACICU2 carrying the *rep*Aci6 gene (Towner et al., 2011).

## Results

### Antimicrobial Susceptibility Testing

The MICs of 14 antimicrobial agents were determined for all *A. baumannii* isolates. Sixty-five (64.4%) and 70 (69.3%) strains were resistant to imipenem and meropenem (**Table 2**). Fifty-six CRAB isolates were recovered and the MICs of imipenem and meropenem were both ranged from 16 to 256 mg/L. Resistance to piperacillin/tazobactam, cefoxitin, ceftazidime, cefotaxime, cefepime, gentamicin, amikacin, minocycline, ciprofloxacin, and trimethoprim/sulfamethoxazole were 71.3% (72/101), 87.1% (88/101), 75.2% (76/101), 85.1% (86/101), 69.3% (70/101), 74.3% (75/101), 62.4% (63/101), 27.7% (28/101), 74.3% (75/101), and 88.1% (89/101), respectively. However, no isolate was resistant to colistin or tigecycline. The MICs of antimicrobial agents for isolates recovered from Renji Hospital were higher than those from Fudan Obstetrics and Gynecology hospital. In addition, 14 isolates from Fudan Obstetrics and Gynecology hospital were all sensitive to imipenem and meropenem (**Table 2**).

**Table 2:** Susceptibility analyses of 101 *A. baumannii* in this study.

Antimicrobial agents	RJ^a^ (*n* = 87)	OG^b^ (*n* = 14)	*N* = 101
	R (*n*, %^c^)	R (*n*, %)	R (*n*, %)
Piperacillin/Tazobactam	72, 82.8%	0, 0	72, 71.3%
Cefoxitin	80, 91.9%	8, 57.1%	83, 87.1%
Ceftazidime	73, 83.9%	3, 21.4%	76, 75.2%
Cefotaxime	83, 95.4%	3, 21.4%	86, 85.1%
Cefepime	70, 80.5%	0, 0	70, 69.3%
Imipenem	65, 74.7%	0, 0	65, 64.4%
Meropenem	70, 80.5%	0, 0	70, 69.3%
Gentamicin	75, 86.2%	0, 0	75, 74.3%
Amikacin	63, 72.4%	0, 0	63, 62.4%
Minocycline	28, 32.2%	0, 0	28, 27.7%
Ciprofloxacin	75, 86.2%	0, 0	75, 74.3%
Trimethoprim-sulfamethoxazole	87, 100%	2, 14.3%	89, 88.1%
Tigecycline	0, 0	0, 0	0, 0
Colistin	0, 0	0, 0	0, 0

*^a^RJ, Renji Hospital Shanghai Jiaotong University School of Medicine; ^b^OG, Obstetrics and Gynecology Hospital of Fudan University; ^c^%R, Resistant percent.*

### Detection of Drug Resistance Genes

PCR screening was performed to detect drug resistant genes in all isolates. The natural oxacillinase, *bla*_OXA-51_-like, were detected in all isolates. And *bla*_OXA-51_-like gene of CRAB strains revealed the presence of *bla*_OXA-66_ by sequencing. The prevalence of *bla*_OXA–23_ and *bla*_AmpC_ gene was 60.4% (61/101) and 51.5% (52/101). Besides, all 56 CRAB isolates contained *bla*_OXA–23_ genes and 54 CRAB isolates contained *bla*_AmpC_. The genes encoding *bla*_OXA-24/58_-like, *bla*_IMP-1_ and *bla*_V IM-1/2_ were not detected in any of the 101 isolates.

### Multilocus Sequence Typing

**FIGURE 2:**
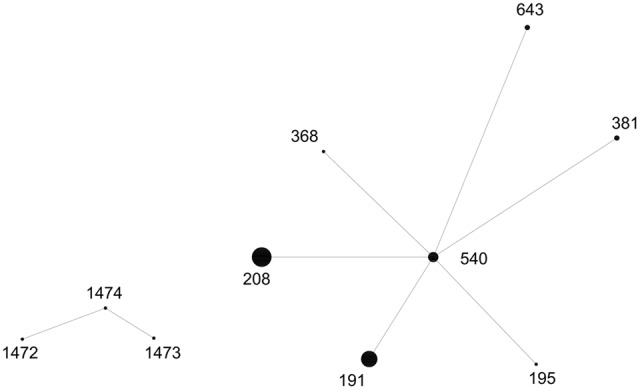
**eBURST analysis results of tested CRAB isolates**. One clonal complexes (CC92) were identified. The size of the dot indicates the number of CRAB isolates.

Fifty-six CRAB isolates were typed by MLST analysis. A total of seven defined STs and three novel STs were identified. ST208, which accounting for the largest proportion (28/56, 50.0%), was the major clonal type, followed by ST191 (12/56, 21.4%), ST540 (7/56, 12.5%), ST381 (2/56, 3.57%), ST643 (2/56, 3.57%), ST195 (1/56, 1.79%), and ST368 (1/56, 1.79%). Additionally, they were single locus variants of gpi gene. eBURST analysis (**Figure 2**) showed that the seven defined STs along with novel STs were all clustered into the same CCs (CC92), which was also commonly referred to as global/international clone II (GCII; Diancourt et al., 2010; Zarrilli et al., 2013). The three novel STs were submitted and have assigned as ST1472, ST1473, and ST1474, respectively.

### Genetic Structure of *bla*_OXA–23_ Gene

The gene encoding *bla*_OXA–23_ of 56 CRAB isolates was located exclusively within the Tn*2006* or Tn*2008* transposons. We found Tn*2008* was almost ubiquitous (54 of 56 isolates). However, the specific region (IS*Aba1-bla*_OXA–23_-IS*Aba1*) for Tn*2006* was only detected in 2 (3.6%) isolates. No IS*Aba4*- *bla*_OXA–23_ was found, indicating there was no Tn*2007* in these CRAB strains, followed by the Tn*2009*.

### Distribution of Plasmid *rep* Genes

GR6 (*rep*Aci6) was the most prevalent and detected in 52 CRAB isolates along with TraC gene, which was identified on plasmid pACICU2 (**Table 3**). GR14 (p4AYE) was found in 29 CRAB isolates, followed by GR8 (Aci9, found in 11 isolates), GR2 (Aci1, 5 isolates), GR4 (Aci4, 3 isolates), and GR12 (p2S1, 1 isolate). The plasmid *rep* gene profiles and CHDL content along with the general typing of 56 CRAB isolates were summarized in **Table 3**.

**Table 3:** Results of plasmid *rep* geng groups of 56 CRAB isolates.

GR group (*rep*Aci)	Isolate(s)	Resistance genes content			Transposons	MLST
		OXA–23	OXA-66	AmpC		
6	60, 80	+	+	+	Tn*2008*	ST208
	25	+	+	+	Tn*2008*	ST191
	36	+	+	+	Tn*2006*	ST381
	56	+	+	-	Tn*2008*	ST208
2	53, 69	+	+	+	Tn*2008*	ST208
	23, 26	+	+	+	Tn*2008*	ST191
6, 8	C3, C26, 19, 21, 22, 24, 30, 33, 50, 55, 57, 61, 63, 82, 91,	+	+	+	Tn*2008*	ST208
	C22,	+	+	+	Tn*2008*	ST1472
	C23,	+	+	+	Tn*2008*	ST1473
	39	+	+	+	Tn*2008*	ST1474
6, 14	18, 28, 32, 37, 40, 41, 59, 76, 88	+	+	+	Tn*2008*	ST191
	C27, 64, 65, 66, 67, 83	+	+	+	Tn*2008*	ST540
	45, 49, 51	+	+	+	Tn*2008*	ST208
	20, 35	+	+	+	Tn*2008*	ST643
	C7	+	+	+	Tn*2008*	ST368
	52	+	+	-	Tn*2008*	ST208
	62	+	+	+	Tn*2006*	ST540
	34	+	+	+	Tn*2008*	ST381
2, 6, 14	48	+	+	+	Tn*2008*	ST195
6, 12, 14	C20	+	+	+	Tn*2008*	ST208
4, 6, 8	71, 84, 85	+	+	+	Tn*2008*	ST208

*+, positive; -, negative.*

## Discussion

The increasing drug resistance of *A. baumannii* has raised a big challenge, especially CRAB strains reported worldwide (Durante-Mangoni et al., 2014). As observed in other literature, here we reported a high prevalence of carbapenem resistance (55.4%) in clinical *A. baumannii* isolates recovered from two hospitals. Most CRAB strains always exhibited high resistance to other antimicrobial agents tested.

OXA-type carbapenems resistance hydrolytic enzymes are common in *A*. *baumannii* isolates. *bla*_OXA-51_-like gene, which shares less than 60% amino acid identity with other OXA-types, was identified in all *A. baumannii* isolates, supporting that *bla*_OXA-51_-like are natural genes in *A*. *baumannii* (**Table 3**). *bla*_OXA-66_ variant might be the most prevalent and found in all 56 CRAB isolates. In general, CRAB strains contained more resistance genes. This probably explains why CRAB strains show much higher MICs than non-CRAB isolates. *bla*_OXA-24_-like β-lactamases was first identified in *A. baumannii* in 1997, which resulted in an outbreak in Spain (Bou et al., 2000). And*bla*_OXA-58_-like gene was first found in France in 2003 (Poirel et al., 2005). Isolates carrying *bla*_OXA-24/58_-like genes were typically resistant to carbapenems. Fortunately, they were barely identified in China. Our results also indicated no *bla*_OXA-24/58_-like nor *bla*_IMP-1_ genes were found. However, due to their plasmid location, the distribution of these genes in *A. baumannii* should be early monitored.

Multilocus Sequence Typing is frequently used for strain phylogeny and global epidemiology. Fifty-six CRAB isolates with occurrence of *bla*_OXA–23_ in our study were typed. The most common ST was ST208 and CC92 was the unique CC clonal group tested. CC92 (GCII) clone outbreaks due to *bla*_OXA–23_-producing *A*. *baumannii* strains have been reported frequently (Fu et al., 2010; He et al., 2011). The discovered of three novel STs indicated that *A. baumannii* isolates were diverse and still clonal expansion. Our e-BURST analysis revealed clonal spread at Renji hospital during a certain period. Hence the carbapenems resistance in *A. baumannii* is increasing. While isolates of the same ST clone reported owning different resistance patterns, the vital spreading of CRAB isolates may have other approaches.

*Acinetobacter baumannii* PCR-based replicon typing assay revealed 52 CRAB isolates owning *rep*Aci6, and the gene encoding TraC was strongly linked with *rep*Aci6 plasmid. No *rep* genes associated with *bla*_OXA-24/58_ were found. *Rep*Aci6 was identified on plasmid pACICU2 in general and TraC protein make pACICU2 transferable (Iacono et al., 2008). In some cases (e.g., isolates Ab18 and Ab64), different MLST genotypes were found to have identical plasmid *rep* gene profiles. In contrast (e.g., isolates Ab18 and Ab23), isolates with the same MLST type were found to have different plasmid *rep* gene profiles (**Table 3**). Hence the plasmid *rep* genes were variable in the process of strain epidemics. *bla*_OXA–23_-like gene was associated with carriage of *rep*Aci6 plasmids as previously described (Towner et al., 2011). With few exceptions, strains grouped in ST208 were associated with pACICU2, pMAC02, and p4AYE. Strains grouped in ST191 and ST540 were associated with carriage of pACICU2 and p4AYE. These removable plasmids carrying CHDLs genes led to spreading among *A. baumannii* strains.

As we mentioned, the spread of carbapenems resistance gene in *A. baumannii* are of great importance. One of the most popular gene *bla*_OXA–23_ was discovered here. Of four transposons reported, Tn*2006* and Tn*2008* were identified in CRAB isolates, both of which were flanked by IS*Aba1*. However, Tn*2007* and Tn*2009* are not seen in agreement with previous researches. It was noted that IS*Aba1* facilitated the mobilization of *bla*_OXA–23_ gene hence allowed Tn*2006* and Tn*2008* to move. These four transposons were observed in plasmids as well as in the chromosome of *A. baumannii* (Nigro and Hall, 2016). Besides, Tn*2006*, Tn*2007*, and Tn*2008* were found in different locations of conjugative *rep*Aci6 group plasmids (Nigro and Hall, 2015). It seemed that the *bla*_OXA–23_ dissemination might due to transposition of mentioned transposons.

Our survey suggested that both clonal spread of a GC2 strain carrying carbapenems resistance genes and the spread of conjugative plasmid among *A. baumannii* strains are responsible for the serious increasing carbapenems resistance. Further understanding of related plasmids may help determine their acquisition, dissemination and regulation among *A. baumannii*.

## Author Contributions

CY conceived the work. YC performed all experiments, analyzed the results, and wrote the manuscript. JG and HZ assisted in antimicrobial sensitivity testing. All authors read and approved the final paper.

## Conflict of Interest Statement

The authors declare that the research was conducted in the absence of any commercial or financial relationships that could be construed as a potential conflict of interest.

## Footnotes

1 http://www.ncbi.nlm.nih.gov/blast
